# Aged Zebrafish as a Spontaneous Model of Cardiac Valvular Disease

**DOI:** 10.1111/acel.70266

**Published:** 2025-10-12

**Authors:** Laura Bevan, Jessica Radford, Helena Urquijo, Joseph Carr, Alice Etheridge, Stephen Cross, Melanie Hezzell, Rebecca J. Richardson

**Affiliations:** ^1^ School of Physiology, Pharmacology & Neuroscience, Faculty of Health and Life Sciences University of Bristol Bristol UK; ^2^ MRC Integrative Epidemiology Unit University of Bristol Bristol UK; ^3^ Population Health Sciences, Bristol Medical School University of Bristol Bristol UK; ^4^ The Wolfson Bioimaging Facility University of Bristol Bristol UK; ^5^ Bristol Veterinary School University of Bristol Bristol UK

**Keywords:** ageing, animal model, cardiovascular diseases, heart, stenosis, zebrafish

## Abstract

Valvular heart disease (VHD) is a highly prevalent age‐associated cardiovascular pathology. VHD can be characterised by stenosis, an increase in valve stiffening commonly due to leaflet calcification, or regurgitation, where backflow of blood can occur as a result of valve remodelling. At present, there is a paucity of spontaneous animal models of valve disease which would aid mechanistic investigations and allow therapeutic screening. Here, we report a spontaneously occurring zebrafish valve disease model, which is associated with natural ageing. Using 2D and 3D morphometric approaches, we identify that aged zebrafish (> 2.5 years old) show greater valve volume and leaflet width/area when compared to young fish (< 1.5 years old). Size and shape changes occur in both the atrioventricular (AV) and bulboventricular valves (BV). Immunofluorescence and histological analyses reveal cellular changes, increased immune cell infiltration and altered distribution of elastin and collagen in aged leaflets, similar to that observed in mammalian clinical samples. Finally, we show that aged zebrafish exhibit cardiac dysfunction associated with valve degeneration. We have demonstrated that this novel zebrafish model of spontaneously occurring age‐related valve degeneration may have utility as a human disease model and could be used to determine mechanistic insights in the future.

## Graphical abstract

Natural ageing in zebrafish results in spontaneous development of valvular degeneration in both the atrioventricular (AV) and bulboventricular (BV) valves. Aged zebrafish valves are larger and exhibit cystic regions, immune cell infiltration, early osteoblast differentiation and increased regurgitation. Zebrafish represent a new valve disease model.

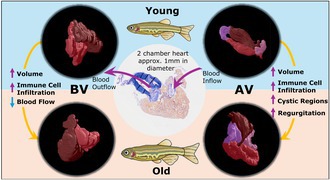

## 1 Introduction

Valvular heart disease (VHD) is a prevalent age‐associated cardiac pathology that affects millions, with studies suggesting it occurs in 2.5% of the adult population (Messika‐Zeitoun et al. 2023; Nkomo et al. 2006; Small et al. 2024). VHD encompasses stenosis, associated with an increase in valve stiffening commonly due to leaflet calcification, or regurgitation, where backflow of blood can occur as a result of valve remodelling. Aortic valve stenosis (AVS)/calcific aortic valve disease (CAVD) is the form of VHD that results in the highest rates of hospitalisation and mortality (Coffey et al. 2016; Iung et al. 2003). Early pathological valve changes are estimated to be present in 40% of those over the age of 75, and this sclerotic stage is associated with a higher risk of subsequent cardiovascular disease (CVD) events, even when accounting for other CVD risk factors (Coffey et al. 2016). It is estimated that approximately 12% of North American and European elderly populations (≥ 75 years) suffer from AVS (Osnabrugge et al. 2013). As disease progresses, fibrotic and calcific remodelling processes cause the thickening and stiffening of the valves and prevent appropriate cusp movement throughout the cardiac cycle (Otto and Prendergast 2014). Ultimately, the obstruction of left ventricular outflow leads to inadequate cardiac output, limited exercise capacity, heart failure and lethal cardiovascular complications (Otto and Prendergast 2014).

Myxomatous mitral valve degeneration/disease (MMVD) differs from calcific disease in that the valve leaflets become thickened, weak and ‘floppy’ rather than stiffened (Neto et al. 2018). This most commonly affects the mitral valve, which accounts for 60% of cases, but can also affect the aortic and tricuspid valves (Neto et al. 2018). The most common complication of MMVD is myxomatous mitral valve prolapse (MMVP), where the valve leaflets bulge back into the atrium, often leading to regurgitation (Neto et al. 2018; Pellerin et al. 2002). Mitral regurgitation, where blood flows back through the valve against the direction of flow, is the most common form of VHD (Nkomo et al. 2006). Severe complications of MMVD include rupture of the chordae tendineae, which tether the leaflets to the papillary muscles, leading to exacerbated regurgitation, MMVP, heart failure and sudden cardiac death (Neto et al. 2018; Pellerin et al. 2002).

The cardiac valve leaflets consist of an interstitium composed of a complex connective tissue rich in extracellular matrix (ECM) elements organised in three layers: the atrialis/ventricularis, spongiosa and fibrosa (Dutta et al. 2021). The interstitial layers are populated by valvular interstitial cells (VICs), which aid valve formation in development and maintenance in adulthood primarily via the secretion of ECM components. The interstitial structure is enveloped in an endothelial layer made up of valvular endothelial cells (VECs). The VEC layer plays a critical role in valve function and maintenance as it provides a physical protective barrier, senses changes in the environment, and maintains tissue homeostasis via paracrine signalling (Dutta et al. 2021; Kraler et al. 2022).

The cellular characteristics of VHD progression differ somewhat between disease types. AVS involves dysfunction of VECs, lipoprotein deposition, immune cell infiltration and calcification of the valve leaflets (Goody et al. 2020). Endothelial dysfunction is induced by shear/mechanical stress and results in changes to nitrogen oxide (NO) signalling, lipid deposition and the behaviour of VICs and immune cells. Reactivation of developmental Notch signalling leads to the transdifferentiation of VECs into endothelial‐derived VICs and, ultimately, into osteoblastic VICs (Hjortnaes et al. 2015; Ma et al. 2020). Conversely, MMVD is characterised by thickening of the spongiosa, fragmented, disrupted and cystic ECM, and accumulation of glycosaminoglycans (Neto et al. 2018; Pellerin et al. 2002). The mechanisms that underlie MMVD include increased transdifferentiation of quiescent VICs into an activated myofibroblastic state, upregulated expression of transforming growth factor‐β (TGF‐β), and dysregulation of the TGF‐β signalling pathway (Tang et al. 2022).

Treatment options for VHD are limited as no pharmacological management options are available, and the only treatment option is valve repair or replacement (Butcher et al. 2011; Nishimura et al. 2016). Early interventions for AVS can include pharmacological control of blood pressure, but more severe disease requires valve commissurotomy/valvuloplasty or, in the most severe cases, complete replacement of the valve itself, commonly via transplant of artificial or xenotransplant/biological valves (Butcher et al. 2011). In recent years, Transcatheter aortic valve implantation/replacement (TAVI/TAVR) has become the surgical treatment of choice (Beerkens et al. 2025). For MMVD, surgical repair of affected leaflets is the preferred option for most patients, and many receive a transcatheter mitral valve intervention, for example, the edge‐to‐edge repair technique (MitraClip) (Nishimura et al. 2016). Full valve replacement remains an option for severe disease, however (Nishimura et al. 2016). Currently, understanding of the precise mechanisms that drive VHD is limited by a paucity of animal models for this disease, which also limits the ability to screen or test new therapeutics. At present, there are no spontaneous small laboratory animal models of VHD, with all current models requiring some form of induction, often through injury, genetic manipulation or pharmacological approaches (Sider et al. 2011).

Adult zebrafish have become a highly valued model of human disease (Dooley and Zon 2000; Lieschke and Currie 2007) due in part to their genetic tractability and high degree of genetic similarity to humans, particularly with regard to disease‐associated genes (Howe et al. 2013). Zebrafish have a two‐chambered heart with one atrium and one ventricle but exhibit cellular composition, heart rate and electrical conduction analogous to humans, making them an attractive model for cardiovascular disease studies (Bournele and Beis 2016). The characterisation of mechanisms underlying cardiac morphogenesis and valve development indicates that these are conserved between mammals and zebrafish (Beis et al. 2005; Hsu et al. 2019). Whilst most zebrafish valve studies have concentrated on developmental morphogenesis, a limited number of adult studies have described analogous valve architecture to mammals consisting of a collagenous fibrosa, a spongiosa rich in proteoglycans and a collagen/elastin‐dense ventricularis/atrialis (O'Donnell and Yutzey 2020; Schulz et al. 2019), making them clinically relevant models for the study of valve disease. Recent reports have also revealed that adult zebrafish are able to regenerate damaged cardiac valves (Bensimon‐Brito et al. 2020) and that some fish may exhibit valve abnormalities (Cooper and Spitsbergen 2015), although the similarity to mammalian clinical phenotypes is not fully determined.

Here, we show that naturally aged zebrafish develop spontaneous changes to valve architecture that resemble those observed in mammalian clinical disease. Aged zebrafish exhibit thickening of the valvular leaflets that are associated with ECM degeneration and upregulation of osteoblastic markers when compared to young adult zebrafish. Both the atrioventricular (AV; analogous to the mitral valve) and bulboventricular valves (BV; analogous to the aortic valve) exhibit phenotypic changes. Additionally, echocardiographic assessments indicate that these changes are associated with altered cardiac function in aged zebrafish. This new model will allow future studies into the genetic and molecular causes of valve disease and will also allow a platform for screening of potential therapeutic avenues.

## 2 Results

**FIGURE 1:**
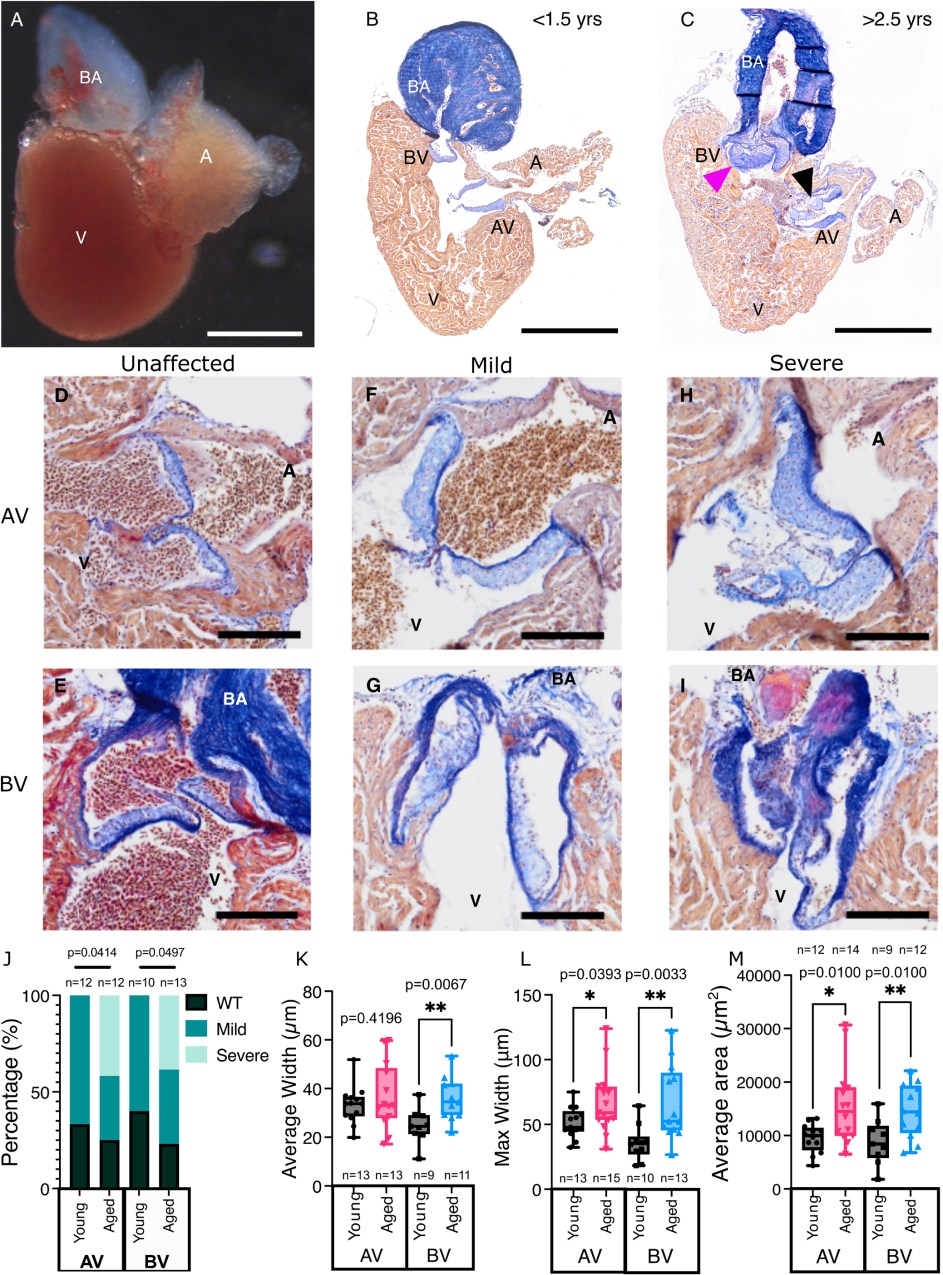
Morphological changes observed in adult zebrafish valves. (A) Image of a dissected heart from a 1‐year‐old zebrafish. (B, C) Overview images of sections of adult zebrafish hearts (< 1.5 years‐B; > 2.5 years‐C) stained with Acid Fuchsin Orange G (AFOG). The atrioventricular (AV) and bulboventricular (BV) valves are indicated. The magenta arrowhead in C indicates thickened leaflets in the BV and the black arrowhead highlights a cystic, nodular region in the AV of an aged zebrafish. (D–I) Higher magnification views of the AV (D, F, H) and BV (E, G, I) indicating examples of phenotypes assigned to the indicated severity for the qualitative scoring shown in J. (J) Qualitive scoring of the AV and BV from young and aged fish. (K–M) Quantification of the average width (K), maximum width (L) and average area (M) of individual leaflets of the AV and BV in young and aged fish. n numbers are indicated on all groups. A, atrium; BA, Bulbus arteriosus; V, ventricle. Statistical analysis: *J*, chi‐squared test; K–M, Welch's *t* tests. Scale bars: A–C = 500 μm; D–I = 100 μm.

Analysis of histological sections stained with Acid Fuchsin Orange G (AFOG) from hearts of wildtype adult zebrafish of mixed ages suggested changes to the size and architecture of both the AV and BV in a subset of fish, with abnormal thickening and large nodular structures often being apparent (Figure 1A–G). We hypothesised that this may be related to the age of the fish. We therefore compared the valves of fish that we designated young (< 1.5 years old) and aged (> 2.5 years old) from the same line and background strain. Similar ages have been designated as young and aged previously (Itou et al. 2012). Outbred zebrafish strains have an average lifespan of 3–4 years (Gerhard et al. 2002), suggesting that 2.5 years is approximately equivalent to ages 52–69 in humans, based on average UK life expectancy. Initially, both the AV and BV were scored for the extent of phenotypic changes (wildtype, mild, severe) (Figure 1B–I). Valves classified as severe were only observed in aged fish (AV—42%, 5/12; BV—38%, 5/13) and only 20%–25% of aged valves were classified as normal (Figure 1J). Of note, the AV of 66% (8/12) and BV of 58% (7/12) young fish already showed mild phenotypic changes (Figure 1J). In young fish, one of the valves was affected in 33.3% (AV: 2/9; BV: 1/9), and both valves in 55.5% (5/9), whereas aged fish 30% (AV: 2/10; BV: 1/10) had one valve affected and 70% (7/10) had both. Morphometric measurements of individual valve leaflets revealed that the maximal width and area were significantly higher in aged fish compared to young for both valves (Figure 1K–M). The average width was only significantly higher in the BV and not the AV of aged fish, indicating a less uniform thickening and appearance (Figure 1K).

Natural ageing was also associated with other cardiac changes. Although the overall size of the ventricle did not alter with age (Figure S1A–C), aged hearts exhibited more interstitial fibrosis as assessed by Masson's Trichrome staining of collagen (Figure S1A′,B′,D), similar to what has been reported in humans with MMVD and during ageing more broadly (Constant Dit Beaufils et al. 2021; Fang et al. 2025). Together, this suggests that natural ageing results in structural cardiac changes that resemble those observed in humans.

**FIGURE 2:**
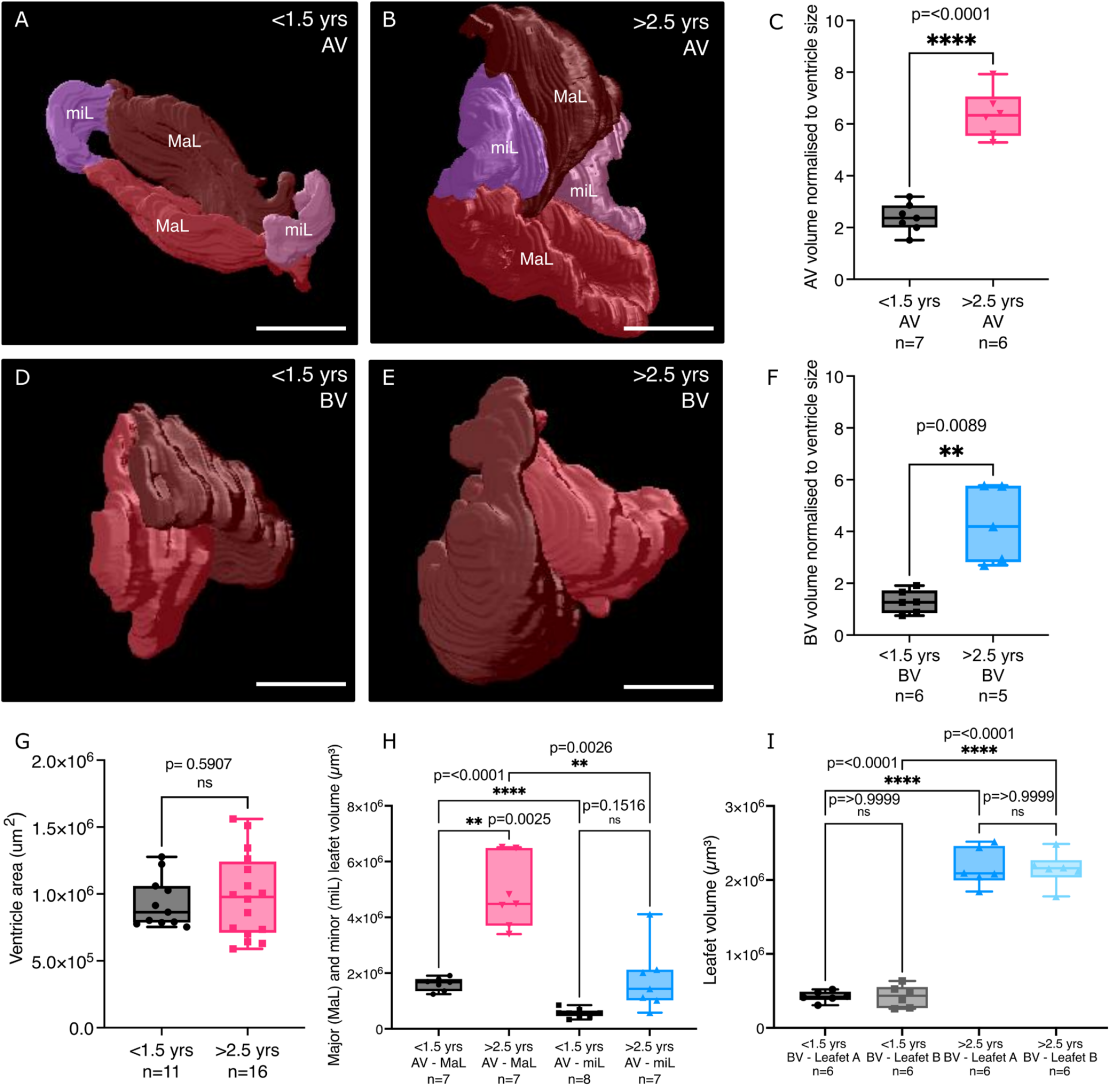
3D render views of adult zebrafish valves. (A–C) 3D render views (A, B) and quantification (C) showing the volume of the entire AV (leaflet volumes combined) in young (A, C) and aged (B, C) fish. (D–F) 3D render views (D, E) and quantification (F) showing the volume of the entire BV in young (D, F) and aged (E, F) fish. In both cases the valve volume is normalised to the ventricular area for each fish. (G) Quantification of ventricular area as measured from confocal imaging of cleared hearts. (H, I) Quantification of the combined volume of the major and minor leaflets of the AV (H) and individual leaflets of the BV (I) in young and aged fish. MaL, major leaflets; miL, minor leaflets. Statistical analysis: C, F, G = Welch's *t* tests; H, I = Brown‐Forsythe and Welch's ANOVA. Scale bars = 100 μm.

We next sought to understand the morphological valve changes further by analysing their shape in 3D (Figure 2, Figure S2 and Videos S1–, S4). We employed tissue clearing (Li et al. 2019) to render the cardiac tissue transparent and used DAPI staining and the inherent autofluorescence of the valve leaflets to create 3D renders of the whole valve using a custom Fiji plugin (Cross et al. 2024; Schindelin et al. 2012). This analysis confirmed that the AV is quadricuspid, consisting of two opposing major leaflets (MaL) and two minor leaflets (miL; Figure S2A–D; Hu et al. 2001). The BV is bicuspid, with two equally sized leaflets (Hu et al. 2001; Figure S2E–H). Comparing the volume of all leaflets combined for each valve, normalised to the ventricular area for each fish, revealed that both the AV and BV were significantly larger in aged fish compared to the AV and BV from young fish, respectively. This enlargement was more pronounced in the AV, following normalisation, and the AV was larger than the BV overall (Figure 2A–F and Videos S1–, S4). Quantification of ventricle area from confocal imaging of cleared hearts further supported our analysis of sections and showed no significant difference in size between young and aged hearts (Figure 2G). Analysis of individual leaflet volume showed that both leaflets of the BV were similarly affected in aged fish when compared to leaflets of young fish (Figure 2I). In the aged AV, however, only the major leaflets showed a significant increase in size when compared to the same leaflets in young fish, suggesting they are more affected than the minor leaflets (Figure 2H).

**FIGURE 3:**
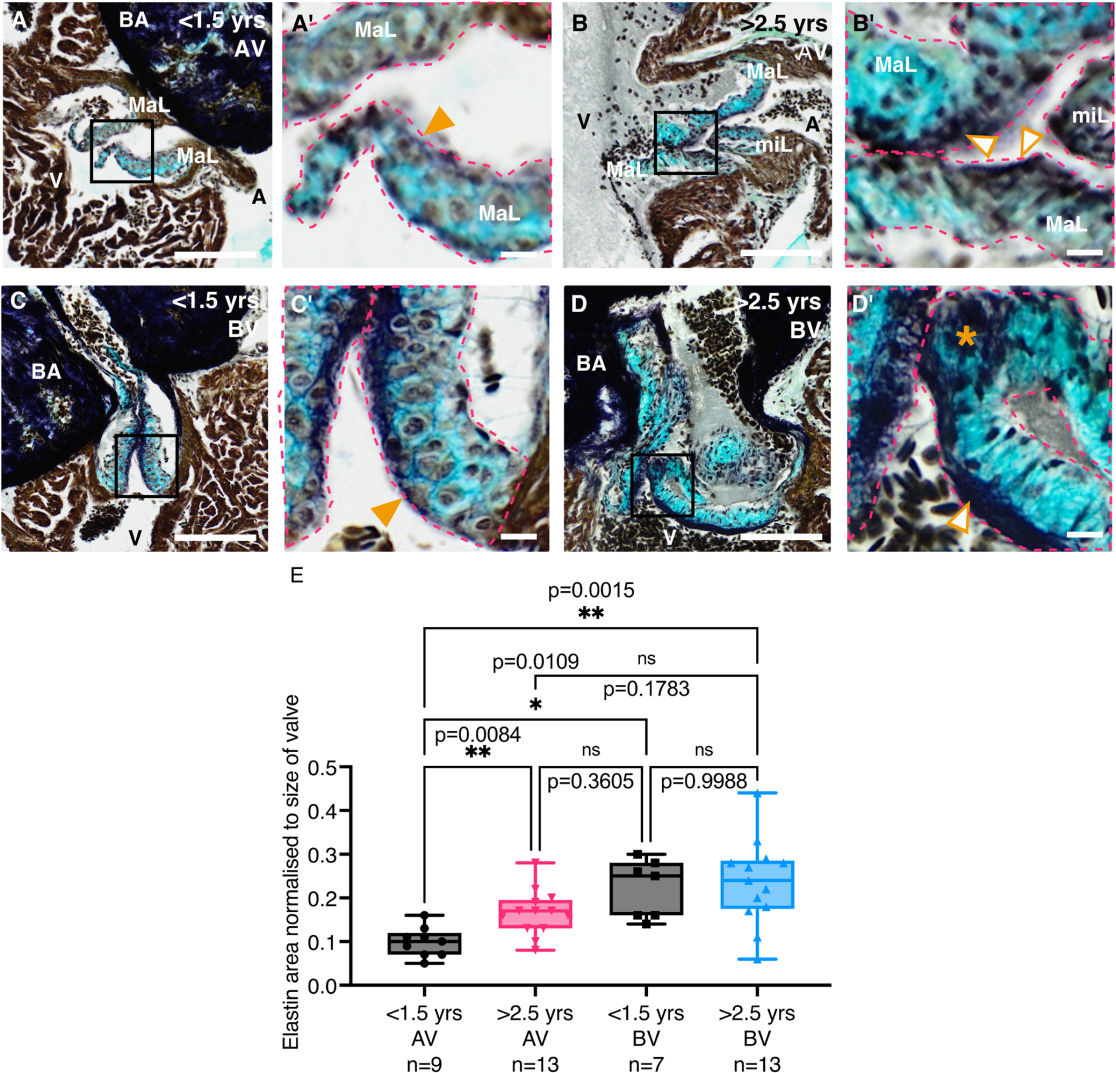
Phenotyping of young and aged zebrafish valves. (A–D) Images of RMP stained valves from young (A, C) and aged (B, D) fish. The AV is shown in A, B and the BV in C, D. (A′–D′) Higher magnification views of the boxed regions in A–D. Orange arrowheads in A′ and C′ denote the thin elastin layer within the atrialis/ventricularis. The open orange arrowheads in B′ and D′ denote the thickened elastin layer in the AV and BV, respectively and the asterisk in D′ indicates a region of disordered elastin. (E) Quantification of the amount of elastin staining, normalised to the area of all leaflets combined, in the AV (left) and BV (right) of young and aged fish. Individual leaflets are outlined with magenta dashed lines in A′–D′. A, atrium; BA, Bulbus arteriosus; MaL, major leaflet; miL, minor leaflet; V, ventricle. Statistical analysis: E, Brown‐Forsythe and Welch's ANOVA. Scale bars: A–D = 100 μm; A′–D′ = 10 μm.

Next, we aimed to characterise the underlying pathological phenotypes of the enlarged valves observed in aged fish (Figure 3 and Figure S3). Although differences in valve structure between zebrafish and mammals have been noted, namely increased cellularity and reduced ECM layering (Schulz et al. 2019), it is also accepted that developmental processes and cellular origins are highly conserved (Beis et al. 2005; Gunawan et al. 2020). To investigate the phenotypic changes observed in the thickened valves of aged fish, we first used histological analyses, immunostaining and confocal imaging of transgenic lines to assess the general architecture of valves of young and aged fish. Whereas both valves from young fish displayed the ordered cellular appearance previously described (Schulz et al. 2019) (Figure S3A,C), the valves of aged fish frequently appeared more disorganised and nodular, with cystic regions exhibiting reduced cellularity (Figure S3B,D). Thickened leaflets from the BV were sometimes observed protruding into the outflow tract in aged fish (Figure S3D). As endothelial denudation is a common finding in mammalian MMVD (Tang et al. 2022), we assessed VEC distribution in young and aged zebrafish using the endothelial marker line Tg(*fli1:EGFP*) (Lawson and Weinstein 2002). Analysis of cleared hearts from transgenic Tg(*fli1:EGFP*) fish revealed a uniform and tightly packed layer of VECs covering the leaflets of the AV and BV in young fish (Figure S3E,G). GFP expression appeared reduced in this layer in the AV of aged fish and less uniform in the BV, with some gaps being apparent (Figure S3F,H).

Russel‐Movat's Pentachrome (RMP) staining, which contains Alcian blue to reveal glycosaminoglycans, strongly labelled the valves in both young and aged fish (Schulz et al. 2019) (Figure 3A–D). RMP also revealed a thin elastin layer within the ventricularis of the valves from young fish (Figure 3A,A′,C,C′), but this appeared thickened and disordered in valves from aged zebrafish (Figure 3B,B′,D,D′). Indeed, quantification of the area of elastin staining, when normalised to the overall size of the valve, revealed significantly higher levels of elastin in the AV of aged fish when compared to young, similar to what has been reported clinically for MVP (Figure 3E) (Sell and Scully 1965; Small et al. 2024). Interestingly, the BV from both age groups contained significantly more elastin than the AV of young fish (Figure 3E). The BV did not show a difference in the amount of elastin between the age groups, although it was highly variable in aged valves, perhaps due to some areas being thickened and some being disordered/degraded (Figure 3E).

**FIGURE 4:**
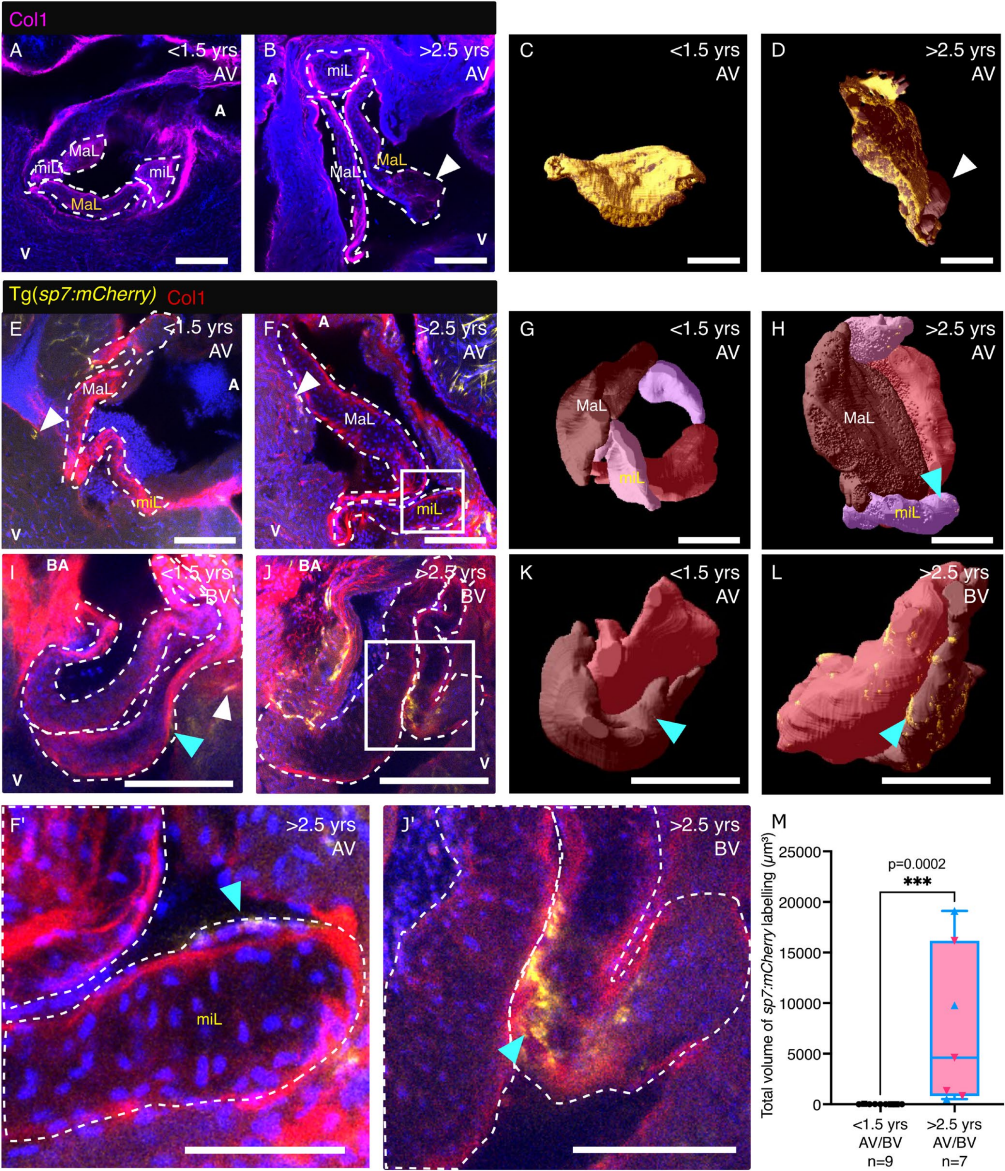
Collagen distribution and osteoblastic differentiation in young and aged zebrafish valves. (A–D) Images of cleared AVs (A, B) and 3D renders of a single leaflet (C, D) from young (A, C) and aged (B, D) zebrafish, labelled with an anti‐Collagen I antibody. The major leaflet (MaL) shown in C, D is marked in yellow on A, B, respectively. White arrowheads in B, D indicate cystic regions devoid of collagen. (E–H) Images (E, F, higher magnification in F′) and 3D renders (G, H) of the cleared AV labelled with an anti‐Collagen I antibody from young (E, G) and aged (F, H) Tg(*sp7:mCherry*) transgenic zebrafish. White arrowheads indicate mCherry+ regions outside the valve. (I–L) Images (I, J, higher magnification in J′) and 3D renders (K, L) of the cleared BV labelled with an anti‐Collagen I antibody from young (I, K) and aged (J, J′, L) Tg(*sp7:mCherry*) transgenic zebrafish. Cyan arrowheads indicate mCherry+ areas within the valve. The boxed regions in F and J indicated the approximate position of F′ and J′. (M) Quantification of the total mCherry+ area within the whole valve of young and aged fish. Due to low sample numbers for individual valves, both the AV and BV are plotted together. Points for aged fish: AV, magenta; BV, blue. Individual leaflets are outlined with white dashed lines in A, B, E, F, I, J, F′, J'. A, atrium; BA, Bulbus arteriosus; MaL, major leaflets; miL, minor leaflets; V, ventricle. Scale bars: A–L = 100 μm; F′, J′ = 50 μm.

To further investigate the observed phenotypic changes, we used immunofluorescence analysis to further investigate both valves in cleared hearts from young and aged transgenic zebrafish in 3D using our custom render (Figure 4 and Videos [Link], [Link], [Link], [Link], [Link], [Link]). Immunofluorescence analysis highlighted the localised distribution of collagen, particularly in the leaflet tethers and the ventricularis of the AV (Figure 4A,B). Analysis of collagen immunofluorescence in 3D further highlighted the disordered nature of the ECM in the AV of aged fish when compared to young (Figure 4A–D, Videos S5, S6) and clearly highlighted cystic regions, which were devoid of Collagen I (arrowheads in Figure 4B,D). To determine if any valvular cells were undergoing osteogenic differentiation, we cleared, labelled with anti‐collagen I antibody and imaged hearts from Tg(*sp7:mCherry*) transgenic zebrafish (Kague et al. 2016). Sp7/Osterix is a well‐established marker of osteoblastic differentiation (Kague et al. 2016; Nakashima et al. 2002) and so mCherry expression in the heart may indicate differentiation to an early osteoblast lineage. Indeed, we observed isolated regions of mCherry expression in young and aged hearts, generally around the base of the valves within the ventricle (Figure 4E,F,I,J). 3D rendering of the entire valve revealed mCherry+ cells within the leaflets of aged fish, predominantly at the base and occasionally at the tip (Figure 4G,H,K,L, Videos S7–, S10). Higher magnification views suggest that the majority of these mCherry+ cells are located on the surface of the leaflets and therefore may be the VECs undergoing transdifferentiation (Figure 4F′,J′). Quantification of the total mCherry+ area within the AV and BV revealed significantly more *sp7* expressing cells in aged valves compared to young (Figure 4M) and these regions were observed in the leaflets of most aged fish examined (5/7 fish total; AV = 2/4 imaged, BV = 4/6 imaged) but at a much lower level in the valves from young fish of the same line (3/10 total; 1/7 AV, 2/3 BV). Together, these data further suggest pathological phenotypes in aged zebrafish valves that resemble clinically observed changes in different forms of VHD.

**FIGURE 5:**
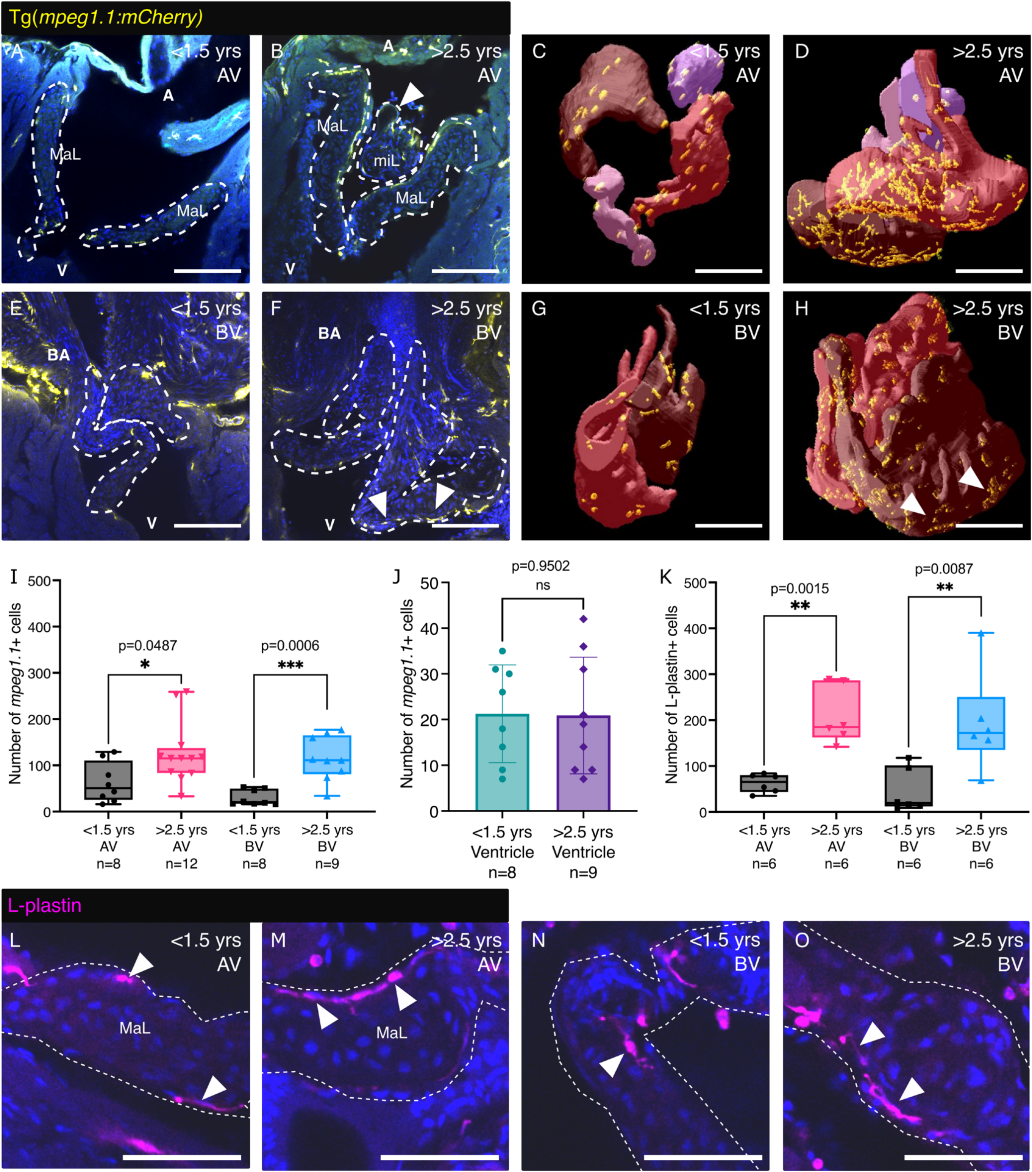
Changes in immune cell number in aged zebrafish valves. (A–D) Images (A, B) and 3D renders (C, D) of the cleared AV from young (A, C) and aged (B, D) Tg(*mpeg1.1:mCherry*) zebrafish. (E–H) Images (E, F) and 3D renders (G, H) of the cleared BV from young (E, G) and aged (F, H) Tg(*mpeg1.1:mCherry*) transgenic zebrafish. White arrowheads indicate macrophages close to cystic regions of the BV in aged zebrafish. (I) Quantification of the number of *mpeg1.1*+ cells associated with the entire AV (left) and BV (right) of young and aged Tg(*mpeg1.1:mCherry*) transgenic zebrafish. (J) Quantification of the number of *mpeg1.1*+ cells in a 200 μm^3^ region of the ventricle (away from the valve) in young and aged Tg(*mpeg1.1:mCherry*) zebrafish. (K) Quantification of the number of L‐plastin+ leukocytes in the AV (left) and BV (right) of young and aged zebrafish. (L–O) Confocal images of cleared valves labelled with an anti‐L‐plastin antibody. Leukocytes can be seen on the surface of all leaflets (arrowheads). Individual leaflets are outlined with white dashed lines in A, B, E, F, L–O. A, atrium; BA, Bulbus arteriosus; MaL, major leaflets; miL, minor leaflets; V, ventricle. Statistical analysis: I, K(BV) = Mann–Whitney tests; J, K(AV) = Welch's *t* test. Scale bars: A–H = 100 μm; M–P = 50 μm.

Immune cell infiltration into the valve leaflets has been linked to the onset and progression of AVS (Goody et al. 2020) and myxomatous degeneration (Kim et al. 2020). Therefore, we next assessed the number of immune cells in the valve using immunostaining with the pan‐leukocyte marker, L‐plastin (Cvejic et al. 2008) and Tg(*mpeg1.1:mCherry*) zebrafish (Figure 5). Clearing, imaging and rendering of valves from young and aged Tg(*mpeg1.1:mCherry*) zebrafish revealed significantly higher numbers of *mpeg1.1*+ cells within the AV and BV of aged fish when compared to young (Figure 5A–I and Videos S11–, S14). This increase was most pronounced in the BV (Figure 5I). The number of L‐plastin+ cells was also increased, and the numbers were higher than those for *mpeg1.1*+ in aged valves, suggesting other cells, beyond those expressing *mpeg1.1*, are present (Figure 5K). The immune cells were often located on the atrialis/ventricularis surfaces of the valve leaflets adjacent to the blood flow, especially in aged fish (Figure 5B,F,L–O), as has been described for murine valves (Hulin et al. 2018).

**FIGURE 6:**
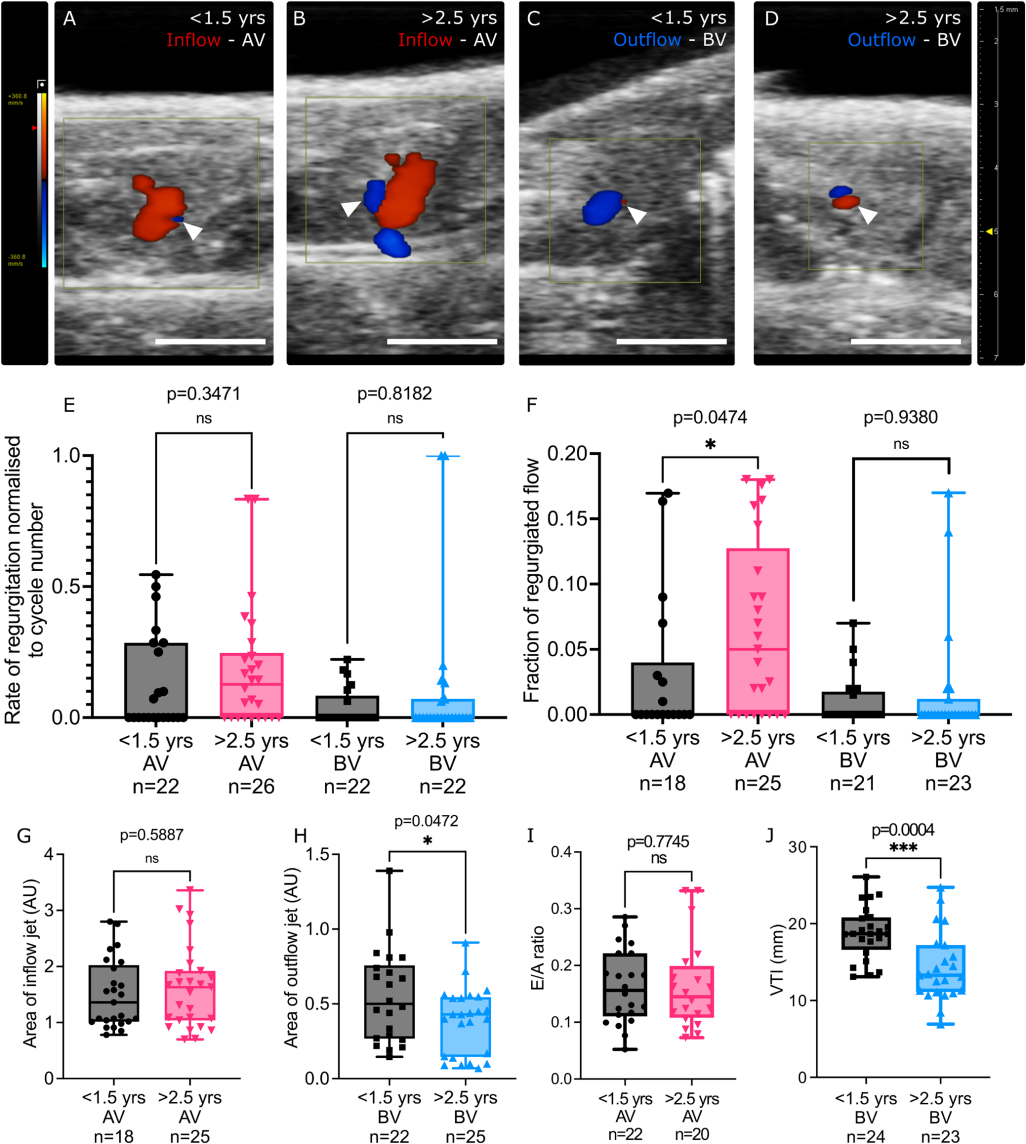
Functional changes in aged zebrafish hearts. (A–D) Example images from colour Doppler analysis revealing the inflow of blood through the AV (red—A, B) and outflow of blood through the BV (blue—C, D) in young (A, C) and aged zebrafish (B, D). Regurgitation is indicated by the opposite colour at the same anatomical region (white arrowheads in A–D). Anterior is to the left. (E, F) Quantification of the rate of regurgitation, normalised to cycle number (E) and the fraction of the regurgitated flow (F) observed at the AV and BV of young and aged zebrafish. (G, H) Area measurements of the maximal flow through the AV (G) and BV (H) in young and aged zebrafish. AU, arbitrary units. (I, J) Quantification of functional parameters of the AV (I) and BV (J) of young and aged zebrafish via pulse wave Doppler echocardiography. Statistical analysis: E, F, G, I = Mann Whitney‐*U* tests, H, J = Welch's *t* tests.

Finally, to evaluate whether the spontaneous abnormalities of the valves in aged zebrafish are accompanied by haemodynamic changes that affect cardiac function, we performed colour Doppler imaging and pulse wave Doppler (PWD) of ventricular inflow and outflow on young and aged zebrafish (Figure S4A–D; Figure 6A–D). Colour Doppler imaging was used to assess regurgitation rates in young and aged fish by measuring the proportion of cardiac cycles displaying retrograde blood flow (Figure 6E). This suggested that regurgitation was common in both age groups and more pronounced in the AV than the BV, with no difference in frequency according to age (Figure 6E). However, the fraction of regurgitated inflow was significantly higher at the AV of aged fish compared to young, but no significant difference in the fraction of regurgitated outflow was observed at the BV (Figure 6F). Interestingly, measurement of the area of the flow jets indicated that there was no difference in inflow between the age groups, but the outflow area was significantly smaller in aged fish compared to young (Figure 6G,H). Pulse wave Doppler indicated that the E wave and A wave velocities and E/A ratio, which in mammals indicate ventricular diastolic function, were not significantly different between the groups (Figure S4E,F; Figure 6E–G). Unlike mammals, the late A wave velocity (resulting from contraction of the atrium) is higher than the E wave velocity (resulting from ventricle relaxation and diastolic filling) in zebrafish (Ho et al. 2002). To address previous reports of sex differences in these measurements (Wang et al. 2017), the E/A ratio was compared between age groups after stratifying by sex, which also revealed no significant differences (Figure S4K). To assess ventricular outflow, the VTI, mean and peak pressure gradients and mean and peak velocities as the blood passes through the BV were analysed (Figure 6H–L; Figure S4G–J). This revealed that all ventricular outflow parameters were significantly reduced in aged fish when compared to young fish (Figure 6H–L; Figure S4G–J), and VTI was only affected by age and not sex (Figure S4L). Together, this functional analysis of young and aged zebrafish hearts suggests specific changes associated with ventricular systolic function that could indicate altered valvular haemodynamic function.

## 3 Discussion

Here, we describe abnormal valvular phenotypes and cardiac dysfunction in wild‐type aged zebrafish. The majority of zebrafish over 2.5 years of age exhibited some form of leaflet thickening in both the AV and BV, with both valves displaying pathological changes. Valve leaflet thickening has been reported as a phenotype observed in clinical VHD (Small et al. 2024) and is reported as a characteristic of both AVS and degenerative valve disease/MVP. Due to their small size, we are able to clear whole zebrafish hearts, image the entire valve and then render both the AV and BV in 3D. This allowed us to confirm that the overall volume of the valve is also increased as well as assess the thickening of individual leaflets. Via phenotypic scoring, over half of the young control zebrafish group (which were all 11–13 months old for this experiment) already exhibited mild phenotypic changes, suggesting that this may be a progressive disease that occurs over time, or that other factors may influence the severity of disease. Unlike other studies (Wang et al. 2017), our echocardiographic assessments did not identify any sex differences in cardiac function within the age groups, suggesting that other factors may influence the heterogeneity observed, particularly within the younger age group. It will be interesting to further investigate this potential progressive degeneration by defining narrower ‘young’ and ‘middle aged’ age groups as this would allow markers of the earliest indicators of disease to be identified.

Our phenotypic investigations into the valve abnormalities observed in aged zebrafish suggest a mixed pathology when compared to mammalian clinical disease. Cystic, nodular regions with reduced cellularity were a common characteristic of severe disease and were especially notable in the AV, resembling myxomatous degenerative VHD (Sell and Scully 1965). The AV also showed the most significant change in volume in aged fish when normalised to the size of the ventricle. However, it was the BV that showed the most marked changes in leaflet thickness, inflammatory infiltration and hemodynamic changes. We also observed changes to VEC coverage and the presence of Sp7/Osterix expression, which indicates early osteogenic differentiation, in most of the aged fish examined and in both the AV and BV, pathologies that are characteristic of AVS (Goody et al. 2020). It is interesting to note that the majority of aged fish examined (70%) exhibited valvular changes in both valves, suggesting that the causes of these phenotypes may be linked. It will be interesting to determine the precise mechanisms that drive disease in one or both cardiac valves in zebrafish.

Our functional echocardiographic assessments suggest that regurgitation is prevalent in both young and aged fish and affects both valves, although it was more common at the AV. In humans, a low level of retrograde blood flow through the mitral valve is very common and not of concern to health (Grayburn and Thomas 2021), and our data suggest this is also the case in zebrafish. Furthermore, our findings indicate no changes in parameters of ventricular inflow into the heart during natural ageing in zebrafish. Pathological regurgitant valves can elicit haemodynamic effects similar to stenotic valves, such as higher peak velocity due to the increase in forward blood flow (Salustri and Almaghrabi 2018), but we did not observe similar changes in aged zebrafish suggesting that the observed regurgitation at the AV does not have overt functional outcomes. This absence of a difference with age (or sex) in parameters of ventricular inflow differs from previous reports of age and sex effects, although the former study only considered ages of 3–12 months (Wang et al. 2017). However, when assessing the area of blood movement, we did observe a significantly greater fraction of regurgitated flow at the AV in aged fish compared to young, suggesting that the observed valvular phenotypes do impact AV closing. No significant difference in regurgitated flow at the BV was observed between ages, although regurgitation was less prevalent at this valve. Further work, potentially by comparing an increased number of narrower age groups and by live imaging of leaflet movement, will help reveal the progression and phenotypic causes of this observed regurgitation in all ages.

The haemodynamic changes seen in aged zebrafish are confined to parameters related to BV valve function and outflow. In AVS patients, stiffening and calcification of the leaflets result in narrowing of the opening to allow blood flow; therefore, this results in higher pressure and velocity through the aortic valve. Our data suggest that, in comparison to younger fish, blood flow is reduced as it passes through the BV. Interestingly, a subpopulation of AVS patients will present with obstruction of flow through the aortic valve but low velocity and low‐pressure gradient, often secondary to other comorbidities or severe AVS (Côté et al. 2017; Sharma et al. 2023). This underlines the complexity of VHD phenotypes and their diagnostic criteria. The increase in collagen labelling observed in the ventricle of aged zebrafish may also contribute to cardiac functional changes. Additionally, we observed a reduced area of ventricular outflow at the BV in aged zebrafish, further suggesting defects in systolic function. Indeed, as VTI is a surrogate readout of systolic function and cardiac output, a reduction in this echocardiography parameter, specifically of the left ventricular outflow tract, has also been shown to be a valuable prognostic indicator for heart failure severity in clinical patients (Tan et al. 2017). Further work will be required to fully determine the extent and precise cause of the changes observed in systolic functional parameters in aged zebrafish.

Our studies of L‐plastin+ leukocytes and Tg(*mpeg1.1:mCherry*) zebrafish suggest an increase in the number of immune cells in both the AV and BV of aged zebrafish when compared to young fish of the same strain. Mpeg1.1 was first described as a macrophage marker in larval zebrafish (Ellett et al. 2011), but we and others have shown that it also labels a population of lymphocytes in adult zebrafish (Ferrero et al. 2020; Moyse and Richardson 2020). Indeed, the valvular L‐plastin+ cell counts are higher than those of *mpeg1.1*+ cells, suggesting infiltration of other immune cell types. It will be interesting, therefore, to assess the relative contributions of different leukocyte populations in the progression of the observed disease phenotype. The role of immune cell infiltration has been established in models of AVS (Goody et al. 2020), but it is only recently that these cells have been shown to be drivers of degenerative valve disease also (Hulin et al. 2018; Kim et al. 2020; Sauls et al. 2015). The observed spontaneous VHD phenotype in almost all naturally aged zebrafish and the data we have provided here, combined with other advantages of zebrafish including their amenability to drug screening, suggest that this will be an ideal model in which to investigate early diagnosis, inflammatory and other disease mechanisms and to screen for potential therapeutics in the future.

## 4 Materials and Methods

### 4.1 Zebrafish Lines

The Tg(*mpeg1.1mCherry*) (Ellett et al. 2011), Tg(*fli1:EGFP*) (Lawson and Weinstein 2002) and Tg(*sp7:mCherry*) (Kague et al. 2016) lines have been described previously. Adult fish used were of mixed sexes and randomly chosen from tanks housing up to 20 individuals. Young fish were classified as being younger than 18 months of age (< 1.5 years) and were typically 7–14 months of age. Aged fish were all 2.6–3.5 years of age (> 2.5 years). For each experiment, matched aged and young fish were of the same line. All lines are maintained on a mixed TL/EKK background. All lines are maintained according to standard procedures, and all animal work is carried out in accordance with UK Home Office and local University of Bristol regulations. Animals were euthanised using the Schedule 1 method of immersion in an overdose of MS‐222 anaesthetic in aquarium water.

### 4.2 Histological and Immunofluorescence Analyses

Standard protocols were used for immunostaining, histology and imaging of stable transgenic fluorescence (Bevan et al. 2020; Moyse et al. 2024). For tissue collection, zebrafish were sacrificed by immersion in 0.4% MS‐222 (Sigma; A5040). Tissues were dissected into ice‐cold PBS, fixed in 4% Paraformaldehyde (PFA) at 4°C, then further washed in PBS prior to immunostaining and tissue clearing and/or embedding in paraffin wax. Antibodies used were rabbit anti‐Collagen I (1:100, Genetex, GTX133063‐GTX), anti‐L‐plastin (1:500; (Cvejic et al. 2008)), chicken anti‐GFP (1:100, Abcam, ab13970) and rat anti‐mCherry (1:100, Thermo Fisher Scientific, M11217), followed by goat anti‐rabbit 647 (1:500, Thermo, A‐21244), goat anti‐chicken 488 (1:500, Thermo, A11039) and goat anti‐rat 555 (1:500, Thermo, A21434), respectively.

### 4.3 Ce3D Tissue Clearing

Tissue clearing was performed using the Ce3D method as previously described (Li et al. 2019; Moyse et al. 2024). Tissues were protected from light at all stages. Whole hearts were fixed in 4% PFA in PBS overnight at 4°C and washed in washing buffer. Immunolabelling and staining steps were performed following fixation and prior to clearing.

### 4.4 Imaging

Imaging of cleared hearts was performed using a Leica TCS SP8 AOBS confocal laser scanning microscope with a 10×/0.4 HC PL APO Dry or 20×/0.75 HC PL APO CS2 Immersion objective. Imaging of histological staining on sections was performed on an Evident (Olympus) VS200 slide scanner microscope with a colour camera and 20×/0.8 Dry objective.

### 4.5 Echocardiography

Zebrafish echocardiography was performed using the Vevo3100 micro‐ultrasound system (FUJIFILM VisualSonics) with a MS550S high‐frequency transducer (FUJIFILM VisualSonics) under the ‘small rodent cardiology’ configuration.

Zebrafish were anaesthetised in 0.025% MS‐222. Fish were transferred to a grooved sponge, positioned ventral side up and submerged in the same anaesthetic solution. Fish were placed on a heated platform to maintain the water temperature throughout imaging. The transducer was mounted at the relevant position and angle for the desired view and immersed in the anaesthetic approximately 1 mm away from the fish. The imaging window was reduced to 6 × 6 mm to improve resolution. Data were collected within 2–5 min following anaesthesia. Individual imaging sessions (at least a week apart) were carried out for pulsed wave Doppler (PWD) of ventricular inflow, PWD of ventricular outflow, and colour Doppler (C‐Mode). Imaging was done in the long axis view (LAX) or the short axis view (SAX). To obtain the LAX view, the transducer was placed parallel to the length of the fish. The SAX view was achieved by rotating the probe 90° so that it was perpendicular to the length of the fish but kept upright. All data were analysed using the Vevo LAB software (5.7.1).

### 4.6 Colour Doppler

Colour Doppler was used to detect blood flow, as well as its direction and intensity, throughout the cardiac cycle. Ventricular inflow is labelled red and ventricular outflow is labelled blue as it flows away from the probe. Videos were captured at a frequency of 32 MHz in the LAX view. Regurgitation was detected by observing blood flowing in the direction opposite to what would be expected for the anatomical position. Instances of regurgitation were counted per valve and normalised for the total number of imaged cardiac cycles. The fraction of regurgitated flow was calculated from the areas of regurgitated and maximal flow in the same cardiac cycle. Area measurements are from an average of at least two cardiac cycles, where possible.

### 4.7 Pulsed Wave Doppler (PWD)

PWD imaging was used to quantify the velocity of blood flow during ventricular inflow and outflow. The region of interest for PWD measurements was identified by finding the cross section of the ventricle where flow was at its strongest with the aid of colour Doppler. PWD imaging was performed at a frequency of 32 MHz and a pulse‐rate frequency (PRF) of between 15 and 25 MHz. PWD imaging was performed in the SAX view as previously described (Fang et al. 2020; Wang et al. 2017). The E wave measures the maximum blood flow velocity resulting from the relaxation of the ventricle (early diastolic filling), whilst the A wave measures the maximum blood flow velocity resulting from the contraction of the atrium (Ho et al. 2002). To obtain the E/A ratio, the E wave (mm/s) was divided by the A wave (mm/s). Parameters of ventricular outflow were measured by measuring the area of the outflow peak, which directly yields the velocity time integral (VTI). The other measurements (peak and mean pressure gradients, and peak and mean velocities) are automatically generated by Vevo LAB.

### 4.8 Image Analysis and Statistics

Images and videos were processed using Fiji (Schindelin et al. 2012). For the 3D rendering of cleared valves, a freely available Modular Image Analysis (MIA; version 1.7.3) workflow automation plugin for Fiji was used (Cross et al. 2024). Briefly, valves were manually segmented in 3D using hand‐annotated regions on key z planes of merged fluorescence channels, with regions on intermediate z planes generated via interpolation. To further refine the segmentation, the fluorescent valve channel was binarised using the Huang method (with a constant multiplication factor of 0.1 applied to systematically reduce the calculated threshold) and the resulting image was used to mask the hand‐annotated valve regions. In addition to valves, features were segmented using custom models for the Weka Trainable Segmentation pixel classification plugin (Arganda‐Carreras et al. 2017). Following binarisation of the resulting probability images, contiguous regions were subdivided using an intensity‐based watershed transform (Legland et al. 2016). Detected features were subject to size‐based filtering, and any identified outside valves were removed from further analysis. Rendering of valves and additional features used the 3DScript plugin (Schmid et al. 2019), with binarised representations of valves and features combined with greyscale fluorescence images as multi‐channel composite image stacks. Composite stacks were interpolated along the *Z*‐axis to give isotropic spatial calibration across *X*, *Y*, and *Z* dimensions.

For quantification of fibrosis from Masson's Trichrome staining, all heart regions beyond the ventricle (atrium, bulbous arteriosus, valves) were manually removed from the image in ImageJ. The percentage of the ventricle positive for collagen was calculated by colour thresholding the whole ventricle and the turquoise labelled regions using ImageJ. For width and area of the valve leaflets from sections, average data indicate measurements from two major leaflets combined. Width measurements were taken at several points across both leaflets, and the average calculated.

In all cases, *n* refers to the number of biological replicates. All experiments were repeated at least twice. Raw data recording and analysis was conducted using GraphPad Prism (v10). All data were checked for outliers via a Grubb's test (alpha = 0.05) and any outliers removed. All data were then checked for normal distribution via a Shapiro–Wilk test (alpha = 0.05) and statistical significance was determined via parametric Welch's *t* tests or nonparametric Mann Whitney‐*U* tests, as appropriate. Details of statistical tests used are provided in figure legends. For qualitative scoring, all images were analysed by the same researcher, blinded to age group. Each valve was scored for degree of thickening of leaflets (mild or severe) and presence of nodular/cystic regions (severe). All quantification was performed blinded to age group. Data is plotted as box and whisker plots with the median and min to max indicated. Individual *p* values are displayed on each graph.

## Author Contributions

L.B., J.R., H.U., J.C. and A.E. designed and performed experiments. S.C. created and advised on custom software. M.H. provided expert advice on experiments. R.J.R. designed the project and supervised the work. All authors wrote and proofread the manuscript.

## Conflicts of Interest

The authors declare no conflicts of interest.

## Supporting information


**Figure S1:** Analysis of ventricle area and fibrosis in young and aged zebrafish hearts. (A, B) Masson's Trichrome staining of hearts from young (< 1.5 years; A) and aged (> 2.5 years; B) zebrafish. (A′, B′) The corresponding ImageJ masks of the regions in A and B, which indicate the amount of turquoise labelled collagen as generated using the colour threshold and analyse particles functions. (C) Quantification of the total ventricle area in young and aged fish. (D) Quantification of the amount of collagen labelling in the ventricle of young and aged fish as assessed by the area of turquoise staining in Masson's Trichrome stained sections. Statistical analysis: C, D = Welch's *t* tests.
**Figure S2:** (A–D) Images of a cleared adult zebrafish heart showing all four leaflets of the AV (A). Coloured outlines of each leaflet are shown in B indicating how leaflet‐specific ROIs were defined (B). Two views of the resulting 3D render with all four leaflets indicated by the colours depicted in B (C, D). (E–H) Similar confocal image (E), ROI depiction (F) and 3D render views (G, H) for the bicuspid BV. MaL, Major leaflet of the AV; miL, minor leaflet of the AV; BA, Bulbus arteriosus; LS, left side, RS, right side. Scale bars = 100 μm.
**Figure S3:** (A–D) Images of the AV (A, B) and BV (C, D) from young (A, A′, C, C′) and aged (B, B′, D, D′) fish stained with AFOG. The boxed regions in A–D denote the approximate position of A′–D′, respectively. Magenta bars in B, C denotes the area that protrudes beyond the annulus in young (C) and aged fish (D). (E–H) Single z positions from confocal imaging of the AV (E, F) and maximum projections of the outflow adjacent surface of the BV (G, H) from young (E, G) and aged (F, H) Tg(*fli1:EGFP*) transgenic zebrafish. The magenta dashed lines outline a single leaflet. Yellow arrowheads in E, F denote the atrialis surface of a MaL of the AV. Open arrowheads in H denote gaps in the VEC layer. MaL, Major leaflet of the AV; miL, minor leaflet of the AV; BA, Bulbus arteriosus. Scale bars: A–D = 100 μm; A′–D′ = 10 μm; E–H = 50 μm.
**Figure S4:** Echocardiography assessment of cardiac function in young and aged zebrafish. (A–D) Example images from colour Doppler (A, B) and (C, D) analysis. Colour Doppler reveals the inflow of blood flow through the AV (A) and outflow of blood through the BV (B). Anterior is to the left. (E–J) Quantification of functional parameters of the AV (E, F) and BV (G–J) of young and aged zebrafish via pulse wave Doppler echocardiography. (K, L) Comparison of E/A ratio (K) and VTI (L) between young and aged fish separated by sex. F, female; M, Male. Statistical analysis: E = Mann Whitney‐*U* test; F, G–J = Welch's *t* tests; K, L = Brown‐Forsythe and Welch's ANOVA tests.

**Video S1:** Rotation of a 3D rendered AV from a young (< 1.5 years) fish. Tissue clearing, imaging and rendering were performed as described in the methods. The major leaflets (MaL) are shown in red and brown and the minor leaflets (miL) are shown in pink and purple.

**Video S2:** Rotation of a 3D rendered AV from an aged (> 2.5 years) fish.

**Video S3:** Rotation of a 3D rendered BV from a young (< 1.5 years) fish. The bicuspid leaflets are labelled in red and brown.

**Video S4:** Rotation of a 3D rendered BV from an aged (> 2.5 years) fish.

**Video S5:** Rotation of a 3D rendered AV from a young (< 1.5 years) fish labelled with an anti‐Collagen I antibody (yellow). One major leaflet (MaL) is shown to increase clarity.

**Video S6:** Rotation of a 3D rendered AV from an aged (> 2.5 years) fish labelled with an anti‐Collagen I antibody (yellow). One major leaflet (MaL) is shown to increase clarity.

**Video S7:** Rotation of a 3D rendered AV from a young (< 1.5 years) Tg(*sp7:mCherry*) fish labelled with an anti‐mCherry antibody (yellow) to reveal regions of early osteoblast differentiation.

**Video S8:** Rotation of a 3D rendered AV from an aged (> 2.5 years) Tg(*sp7:mCherry*) fish labelled with an anti‐mCherry antibody (yellow) to reveal regions of early osteoblast differentiation.

**Video S9:** Rotation of a 3D rendered BV from a young (< 1.5 years) Tg(*sp7:mCherry*) fish labelled with an anti‐mCherry antibody (yellow) to reveal regions of early osteoblast differentiation.

**Video S10:** Rotation of a 3D rendered BV from an aged (> 2.5 years) Tg(*sp7:mCherry*) fish labelled with an anti‐mCherry antibody (yellow) to reveal regions of early osteoblast differentiation.

**Video S11:** Rotation of a 3D rendered AV from a young (< 1.5 years) Tg(*mpeg1.1:mCherry*) fish labelled with an anti‐mCherry antibody (yellow) to reveal macrophages.

**Video S12:** Rotation of a 3D rendered AV from an aged (> 2.5 years) Tg(*mpeg1.1:mCherry*) fish labelled with an anti‐mCherry antibody (yellow) to reveal macrophages.

**Video S13:** Rotation of a 3D rendered BV from a young (< 1.5 years) Tg(*mpeg1.1:mCherry*) fish labelled with an anti‐mCherry antibody (yellow) to reveal macrophages.

**Video S14:** Rotation of a 3D rendered BV from an aged (> 2.5 years) Tg(*mpeg1.1:mCherry*) fish labelled with an anti‐mCherry antibody (yellow) to reveal macrophages.

## Data Availability

The data that support the findings of this study are available in the Supporting Information of this article.
