# Supplementary material for: Aged Zebrafish as a Spontaneous Model of Cardiac Valvular Disease
Source: Aging Cell. 2025 Oct 12;24(12):e70266. doi: 10.1111/acel.70266 (PMC12686566; doi:10.1111/acel.70266)
Supplement: Supplementary file 1 — Figure S1: Analysis of ventricle area and fibrosis in young and aged zebrafish hearts. (A, B) Masson's Trichrome staining of hearts from young (< 1.5 years; A) and aged (> 2.5 years; B) zebrafish. (A′, B′) The corresponding ImageJ masks of the regions in A and B, which indicate the amount of turquoise labelled collagen as generated using the colour threshold and analyse particles functions. (C) Quantification of the total ventricle area in young and aged fish. (D) Quantification of the amount of collagen labelling in the ventricle of young and aged fish as assessed by the area of turquoise staining in Masson's Trichrome stained sections. Statistical analysis: C, D = Welch's t tests. Figure S2: (A–D) Images of a cleared adult zebrafish heart showing all four leaflets of the AV (A). Coloured outlines of each leaflet are shown in B indicating how leaflet‐specific ROIs were defined (B). Two views of the resulting 3D render with all four leaflets indicated by the colours depicted in B (C, D). (E–H) Similar confocal image (E), ROI depiction (F) and 3D render views (G, H) for the bicuspid BV. MaL, Major leaflet of the AV; miL, minor leaflet of the AV; BA, Bulbus arteriosus; LS, left side, RS, right side. Scale bars = 100 μm. Figure S3: (A–D) Images of the AV (A, B) and BV (C, D) from young (A, A′, C, C′) and aged (B, B′, D, D′) fish stained with AFOG. The boxed regions in A–D denote the approximate position of A′–D′, respectively. Magenta bars in B, C denotes the area that protrudes beyond the annulus in young (C) and aged fish (D). (E–H) Single z positions from confocal imaging of the AV (E, F) and maximum projections of the outflow adjacent surface of the BV (G, H) from young (E, G) and aged (F, H) Tg(fli1:EGFP) transgenic zebrafish. The magenta dashed lines outline a single leaflet. Yellow arrowheads in E, F denote the atrialis surface of a MaL of the AV. Open arrowheads in H denote gaps in the VEC layer. MaL, Major leaflet of the AV; miL, minor leaflet of the AV; BA, B [file ACEL-24-e70266-s008.docx]

**Supplementary Material**


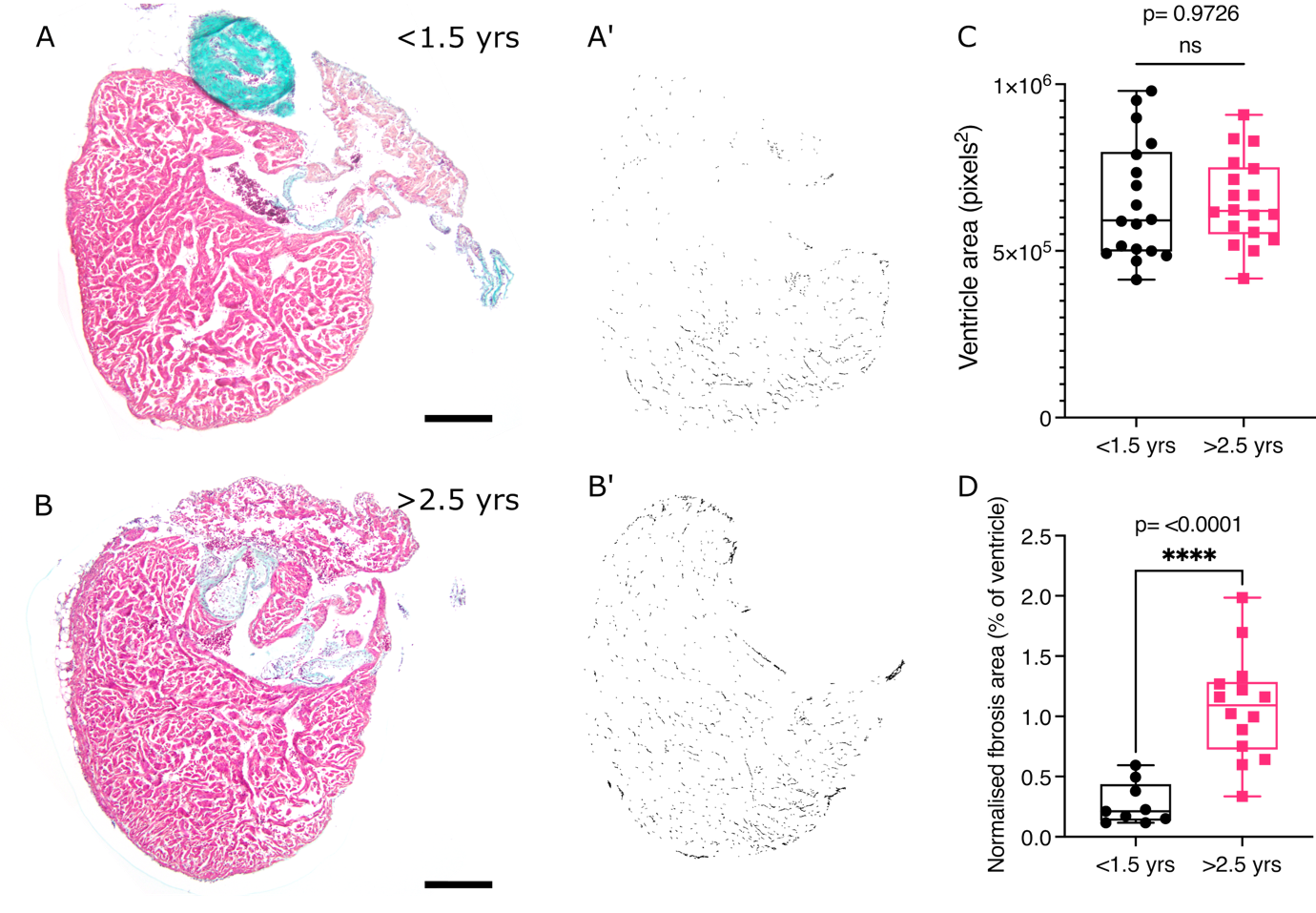


**Supplementary Figure 1 – Analysis of ventricle area and fibrosis in young and aged zebrafish hearts.** (A,B) Masson's Trichrome staining of hearts from young (<1.5 yrs; A) and aged (>2.5 yrs; B) zebrafish. (A’,B’) The corresponding ImageJ masks of the regions in A and B, which indicate the amount of turquoise labelled collagen as generated using the colour threshold and analyse particles functions. (C) Quantification of the total ventricle area in young and aged fish. (D) Quantification of the amount of collagen labelling in the ventricle of young and aged fish as assessed by the area of turquoise staining in Masson's Trichrome stained sections. Statistical analysis: C,D = Welch’s t tests.


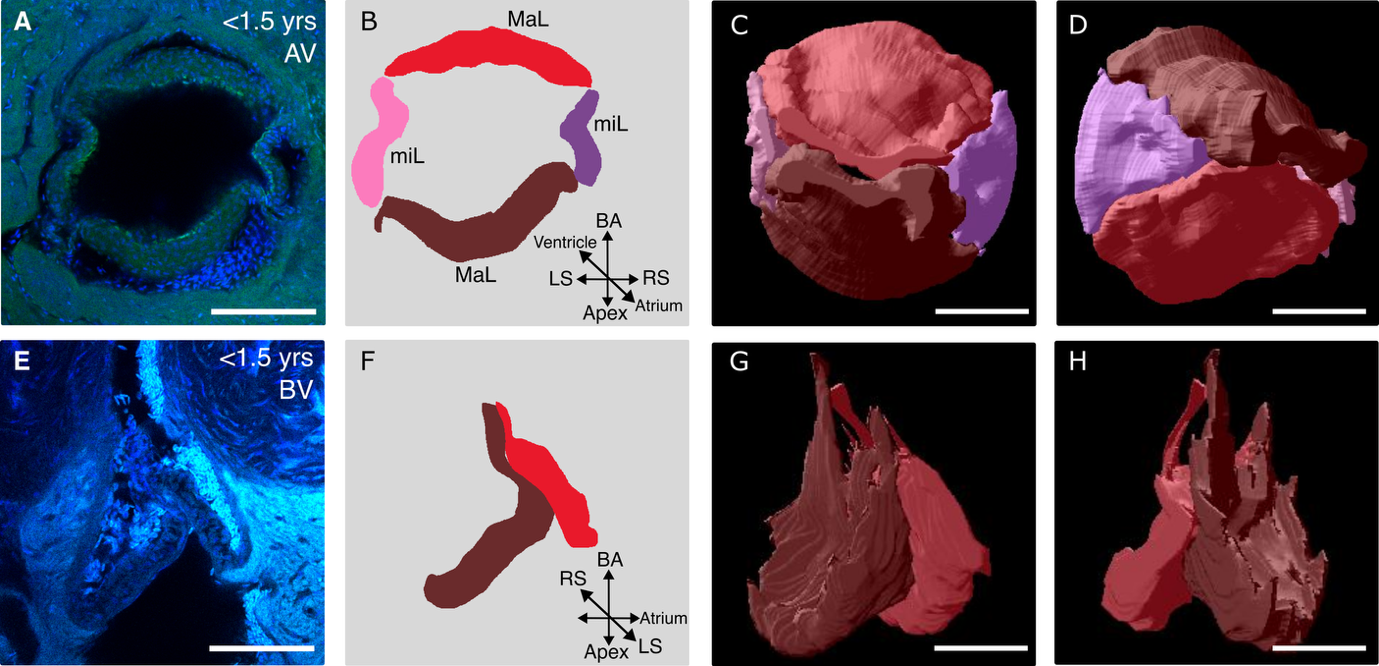


**Supplementary Figure 2 –** (A-D) Images of a cleared adult zebrafish heart showing all four leaflets of the AV (A). Coloured outlines of each leaflet are shown in B indicating how leaflet-specific ROIs were defined (B). Two views of the resulting 3D render with all four leaflets indicated by the colours depicted in B (C,D). (E-H) Similar confocal image (E), ROI depiction (F) and 3D render views (G,H) for the bicuspid BV. MaL = Major leaflet of the AV; miL = minor leaflet of the AV; BA = Bulbus arteriosus; LS = left side, RS = right side. Scale bars = 100 µm.

**
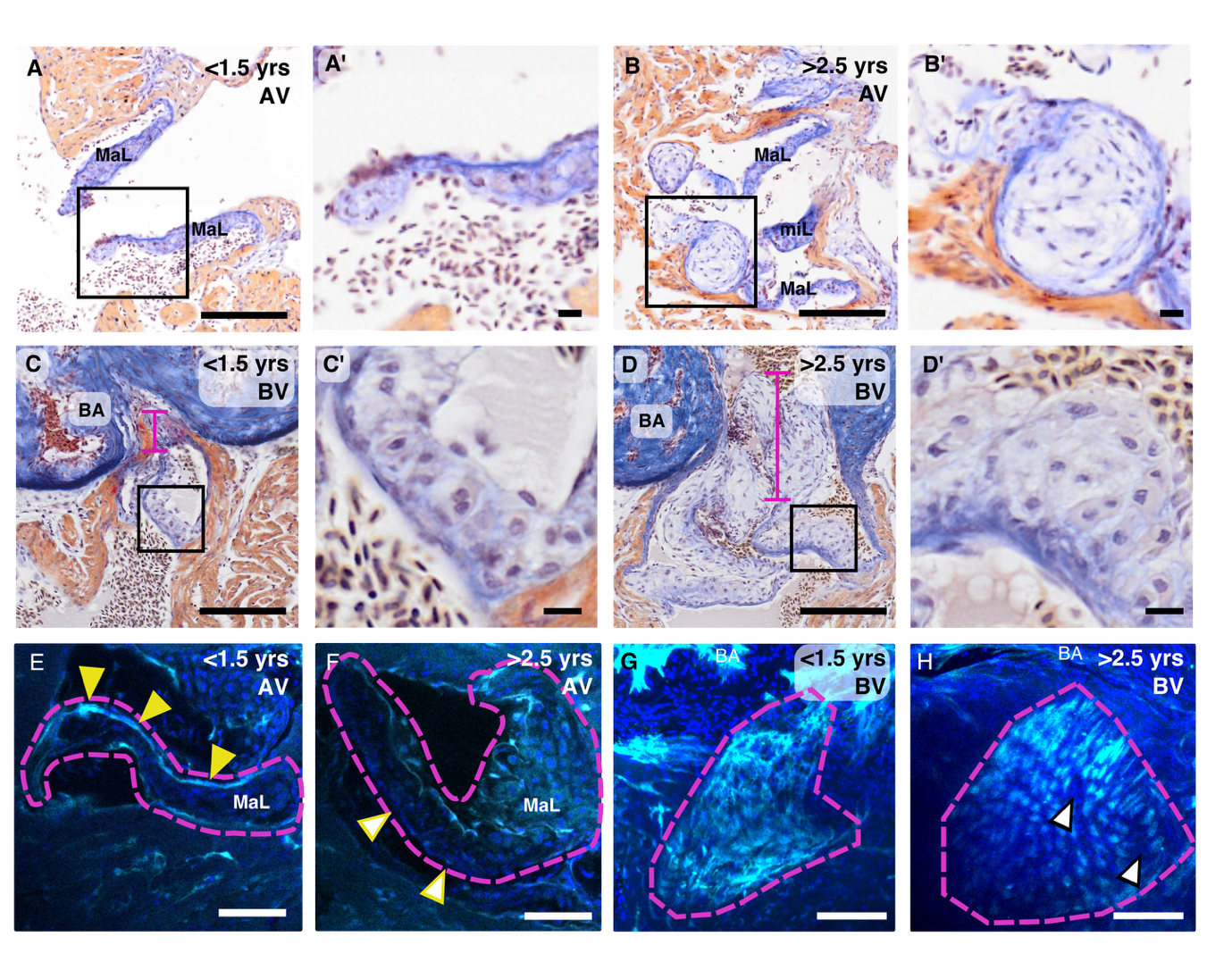
**

**Supplementary Figure 3 –** (A-D) Images of the AV (A,B) and BV (C,D) from young (A,A’,C,C’) and aged (B,B’,D,D’) fish stained with AFOG. The boxed regions in A-D denote the approximate position of A’-D’, respectively. Magenta bars in B, C denotes the area that protrudes beyond the annulus in young (C) and aged fish (D). (E-H) Single z positions from confocal imaging of the AV (E,F) and maximum projections of the outflow adjacent surface of the BV (G,H) from young (E,G) and aged (F,H) Tg(*fli1:EGFP*) transgenic zebrafish. The magenta dashed lines outline a single leaflet. Yellow arrowheads in E,F denote the atrialis surface of a MaL of the AV. Open arrowheads in H denote gaps in the VEC layer. MaL = Major leaflet of the AV; miL = minor leaflet of the AV; BA = Bulbus arteriosus. Scale bars: A-D = 100 µm; A’-D’ = 10 µm; E-H = 50 µm.

**
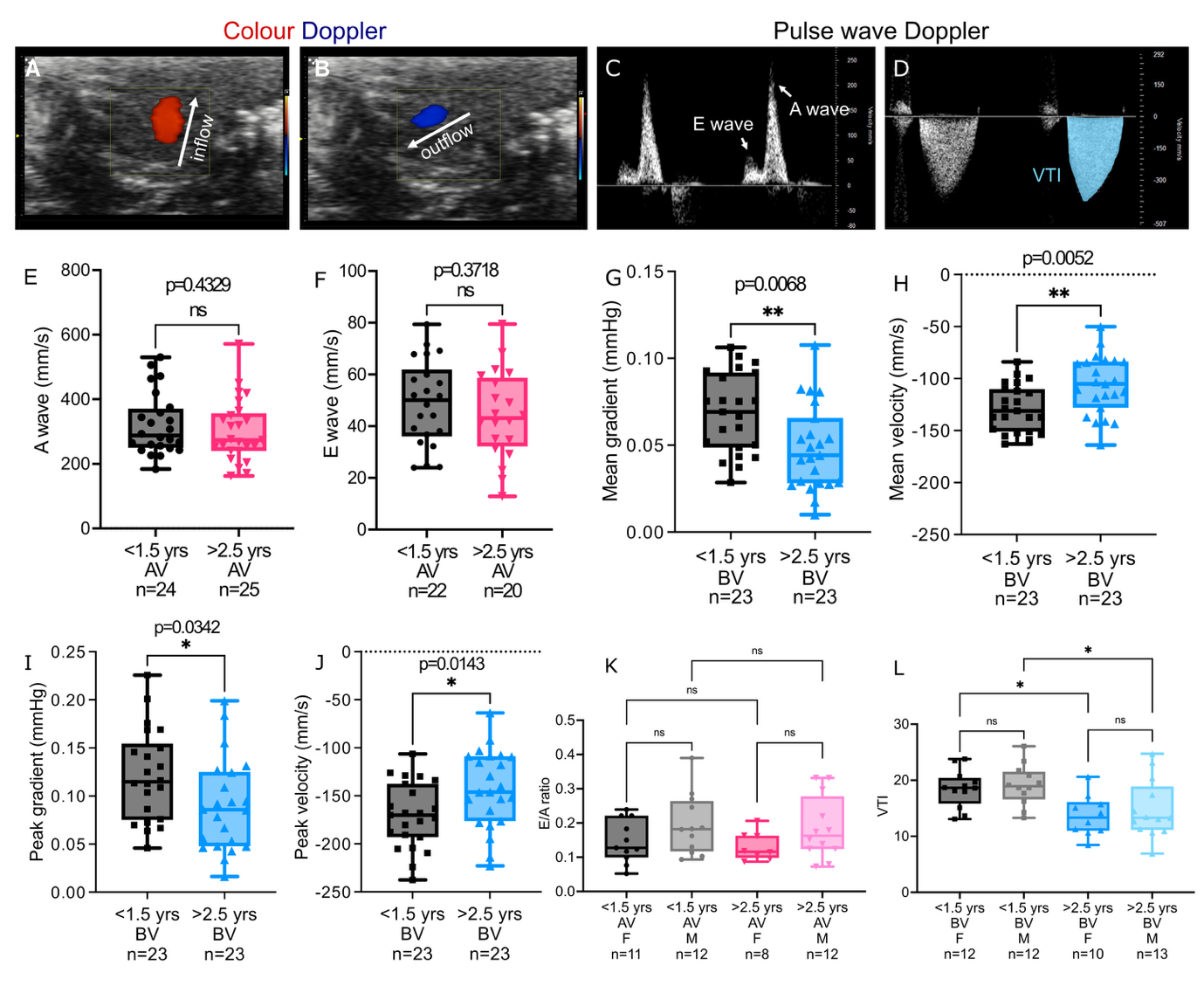
**

**Supplementary Figure 4 – Echocardiography assessment of cardiac function in young and aged zebrafish.** (A-D) Example images from colour Doppler (A,B) and (C,D) analysis. Colour Doppler reveals the inflow of blood flow through the AV (A) and outflow of blood through the BV (B). Anterior is to the left. (E-J) Quantification of functional parameters of the AV (E,F) and BV (G-J) of young and aged zebrafish via pulse wave Doppler echocardiography. (K,L) Comparison of E/A ratio (K) and VTI (L) between young and aged fish separated by sex. F = female, M = Male. Statistical analysis: E = Mann Whitney-U test; F,G-J = Welch’s t tests; K,L = Brown-Forsythe and Welch’s ANOVA tests.

**Supplementary video legends**

**Supplementary video 1:** Rotation of a 3D rendered AV from a young (<1.5 yrs) fish. Tissue clearing, imaging and rendering were performed as described in the methods. The major leaflets (MaL) are shown in red and brown and the minor leaflets (miL) are shown in pink and purple.

**Supplementary video 2:** Rotation of a 3D rendered AV from an aged (>2.5 yrs) fish.

**Supplementary video 3:** Rotation of a 3D rendered BV from a young (<1.5 yrs) fish. The bicuspid leaflets are labelled in red and brown.

**Supplementary video 4:** Rotation of a 3D rendered BV from an aged (>2.5 yrs) fish.

**Supplementary video 5:** Rotation of a 3D rendered AV from a young (<1.5 yrs) fish labelled with an anti-Collagen I antibody (yellow). One major leaflet (MaL) is shown to increase clarity.

**Supplementary video 6:** Rotation of a 3D rendered AV from an aged (>2.5 yrs) fish labelled with an anti-Collagen I antibody (yellow). One major leaflet (MaL) is shown to increase clarity.

**Supplementary video 7:** Rotation of a 3D rendered AV from a young (<1.5 yrs) Tg(*sp7:mCherry*) fish labelled with an anti-mCherry antibody (yellow) to reveal regions of early osteoblast differentiation.

**Supplementary video 8:** Rotation of a 3D rendered AV from an aged (>2.5 yrs) Tg(*sp7:mCherry*) fish labelled with an anti-mCherry antibody (yellow) to reveal regions of early osteoblast differentiation.

**Supplementary video 9:** Rotation of a 3D rendered BV from a young (<1.5 yrs) Tg(*sp7:mCherry*) fish labelled with an anti-mCherry antibody (yellow) to reveal regions of early osteoblast differentiation.

**Supplementary video 10:** Rotation of a 3D rendered BV from an aged (>2.5 yrs) Tg(*sp7:mCherry*) fish labelled with an anti-mCherry antibody (yellow) to reveal regions of early osteoblast differentiation.

**Supplementary video 11:** Rotation of a 3D rendered AV from a young (<1.5 yrs) Tg(*mpeg1.1:mCherry*) fish labelled with an anti-mCherry antibody (yellow) to reveal macrophages.

**Supplementary video 12:** Rotation of a 3D rendered AV from an aged (>2.5 yrs) Tg(*mpeg1.1:mCherry*) fish labelled with an anti-mCherry antibody (yellow) to reveal macrophages.

**Supplementary video 13:** Rotation of a 3D rendered BV from a young (<1.5 yrs) Tg(*mpeg1.1:mCherry*) fish labelled with an anti-mCherry antibody (yellow) to reveal macrophages.

**Supplementary video 14:** Rotation of a 3D rendered BV from an aged (>2.5 yrs) Tg(*mpeg1.1:mCherry*) fish labelled with an anti-mCherry antibody (yellow) to reveal macrophages.
